# Intra- and inter-specific differences of upper thermal limits in the sand darters (Percidae: *Ammocrypta*)

**DOI:** 10.1093/conphys/coag055

**Published:** 2026-08-10

**Authors:** B L Firth, A R Holsopple, B R Kuhajda, A L George, D A R Drake, M Power

**Affiliations:** Department of Biology, University of Waterloo, 200 University Ave, Waterloo, ON N2L 3G1, Canada; Tennessee Aquarium Conservation Institute, 175 Baylor School Rd, Chattanooga, TN 37405, USA; New York State Department of Environmental Conservation, 625 Broadway, Albany, NY 12233, USA; Tennessee Aquarium Conservation Institute, 175 Baylor School Rd, Chattanooga, TN 37405, USA; Tennessee Aquarium Conservation Institute, 175 Baylor School Rd, Chattanooga, TN 37405, USA; Great Lakes Laboratory for Fisheries and Aquatic Sciences, Fisheries and Oceans Canada, 867 Lakeshore Rd, Burlington, ON L7S 1A1, Canada; Department of Biology, University of Waterloo, 200 University Ave, Waterloo, ON N2L 3G1, Canada

**Keywords:** Agitation temperature, critical thermal maximum, species at risk, surrogate species

## Abstract

Intra- and inter-specific differences in upper thermal limits, such as thermal tolerance, can impact conservation decision-making, yet values are typically obtained from a single population. In this study, we assessed the intra- and inter-specific variation of critical thermal maximum (CT_max_) and agitation temperature (T_ag_) among two eastern sand darter (*Ammocrypta pellucida*) populations (intra-specific) and five species of sand darters (genus *Ammocrypta*; inter-specific). The *Ammocrypta* genus is a monophyletic clade of ecologically similar benthic fishes demonstrating high niche conservatism. Field based CT_max_ trials were conducted stream-side across seasons (April–August 2019 and 2023) to encompass a range of ambient water temperatures (12–28°C). No intra-specific differences in CT_max_ or T_ag_ between eastern sand darter populations were observed, but population was important in predicting T_ag_. Minimal inter-specific differences were observed within *Ammocrypta* with only eastern and naked sand darters (*A. pellucida* and *Ammocrypta beanii*) having consistent differences in CT_max_, while the southern sand darter (*Ammocrypta meridiana*) had lower acclimation capacity. Overall, study results provide a better understanding of thermal tolerance differences within *Ammocrypta* and underline the need for estimates of population- and species-specific tolerances for conservation-based decisions regarding species capacities to withstand climate change.

## Introduction

Climate change and anthropogenic stressors, such as urbanization, are changing the temperature regime in freshwater ecosystems (van  Vliet  *et al.*, 2013; IPCC, 2023). As ectotherms, fishes are particularly vulnerable to increasing water temperatures since temperature governs key physiological processes (e.g. metabolism, growth; McNab, 2002). Consequently, the temperature-dependent nature of numerous traits has shaped species’ niches and their subsequent geographic distribution (Schulte  *et al.*, 2011; Sunday  *et al.*, 2012). To maintain their preferred thermal niche, species may shift their distribution with changes in water temperature (Sunday  *et al.*, 2012; Freeman  *et al.*, 2018). Many freshwater fishes, especially small-bodied stream species, have limited dispersal opportunities (Clavel  *et al.*, 2011; Blanchet  *et al.*, 2013; Kneitel, 2018; LeMoine  *et al.*, 2020), which can cause species to experience sub-optimal conditions that can negatively impact locomotion, reproduction and growth (Fry, 1971; Ficke  *et al.*, 2007). Such negative effects can reduce fitness and will eventually lead to population declines and potential extirpation (Fry, 1971; Ficke  *et al.*, 2007). In the short term, fishes can adjust their physiology to withstand increases in temperature. Thus, determining how temperature-dependent physiology changes with temperature is important for defining a species’ niche and the potential to survive within watersheds under climate change.

Two key metrics for understanding a species’ thermal niche are agitation temperature (T_ag_) and critical thermal maximum (CT_max_; Fry, 1971; Elliott, 1981). Agitation temperature is a sub-lethal behavioural metric, typically observed as rapid swimming and digging, defining the point at which fish begin to actively seek thermal refuge when exposed to increasing temperature (McDonnell  and  Chapman, 2015; Dodsworth  *et al.*, 2025). Agitation temperature has been associated with peak heart rate and cellular stress (e.g. increased transcript abundance and enzyme activity (Bouyoucos  *et al.*, 2023; Durhack  *et al.*, 2025)), implying that exposure to T_ag_ could be associated with reductions in fitness due to thermal stress. Therefore, the temperature below T_ag_ indicates some degree of thermal safety before behavioural alteration and sub-lethal temperature effects could occur. In contrast, CT_max_ is a near-lethal endpoint where physiological dysfunction occurs and beyond which mortality in the wild is expected if temperatures persist at or above CT_max_ (Cowles  and  Bogert, 1944; Hutchison, 1961). Fishes can demonstrate phenotypic plasticity in both metrics (Beitinger  and  Bennett, 2000; Potts  *et al.*, 2021; Reemeyer  *et al.*, 2024). The degree of phenotypic plasticity, which can be measured by the acclimation response ratio (ARR; the change in upper thermal limits relative to the change in water temperature), can be used to estimate how long a species may persist before loss of thermal safety (Morley  *et al.*, 2019). A larger ARR indicates a longer timeframe before environmental temperatures reach T_ag_ or CT_max_ (Morley  *et al.*, 2019). The phenotypic plasticity of CT_max_ and T_ag_, therefore, plays a key role in understanding species resilience to climate change. However, because plasticity varies among species and populations (Walsh  *et al.*, 1997; Fangue  *et al.*, 2006; McDermid  *et al.*, 2013; Reemeyer  *et al.*, 2024), determining the degree of plasticity within and among species is helpful for informing conservation decisions related to population responses to thermal stress.

Among species, fishes can demonstrate substantial differences in thermal response, even when they have close phylogenetic relationships, similar life history, and occupy similar habitats (Hlohowskyj  and  Wissing, 1985; Walsh  *et al.*, 1997; Reemeyer  *et al.*, 2024). Congeners, as organisms in the same genus, may have adapted to different microhabitat conditions, which likely impacts their thermal tolerance and metabolic rate (Walsh  *et al.*, 1997; Wenger, 2008; Campos  *et al.*, 2017; Reemeyer  *et al.*, 2024). For example, the pugnose and blackchin shiner (*Miniellus anogenus and Miniellus heterodon*), co-occurring species that occupy the same general aquatic habitat, have different CT_max_ and T_ag_ (Reemeyer  *et al.*, 2024). Most congener papers only compare a few species that may not even be within the same clade without considering variation in the whole genus (Hlohowskyj  and  Wissing, 1985; Walsh  *et al.*, 1997; Campos  *et al.*, 2017; Reemeyer  *et al.*, 2024). Thus, there is a need to assess inter-specific differences in thermal responses among species that have close phylogenetic relationships and occupy similar habitats. Determining the similarity in responses can help to determine whether and how related species could serve as surrogate species for conservation decisions.

In addition to the potential for inter-specific differences, intra-specific population differences raise the possibility that thermal responses can vary across a species’ range. Population-level differences are common, typically arising from local adaptation to temperature differences across a species’ range, whereby warm-exposed individuals have greater thermal tolerance (Fangue  *et al.*, 2006; McKenzie  *et al.*, 2021; Zillig  *et al.*, 2023; Stewart  *et al.*, 2024). Consequently, assuming a single thermal tolerance for a species could lead to inaccurate conservation decisions. Furthermore, species with a large geographic range will not experience the same environmental change across their range (Sunday  *et al.*, 2012, 2019; Ruthsatz  *et al.*, 2024). Thus, determining among-population (i.e. intra-specific) differences in thermal tolerance is essential for determining whether some populations may have greater susceptibility to thermal stress and may be more susceptible to extinction risk from climate change.

**Figure 1:**
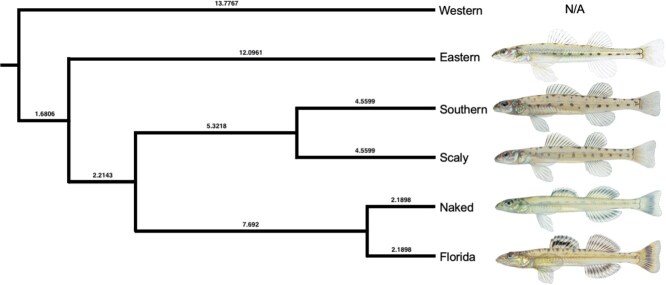
Phylogeny of the *Ammocrypta* clade, western sand darter (*A. clara*), eastern sand darter (*A. pellucida*), southern sand darter (*A. meridiana*), scaly sand darter (*A. vivax*), naked sand darter (*A. beanii*) and Florida sand darter (*A. bifascia*) using data obtained from Near  *et al.*  (2011) with images created by and used with permission from Joseph R. Tomelleri. A picture for western sand darter is not available; however, the species is similar to eastern sand darter but with a spine on the opercula.

**Figure 2:**
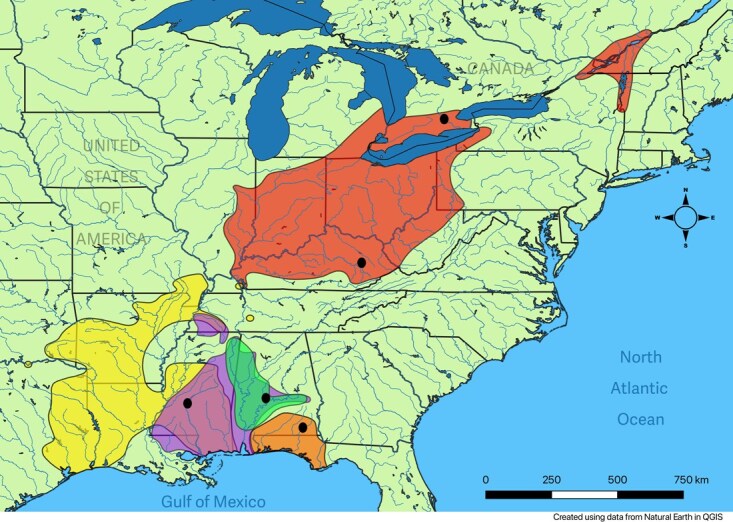
Geographic range of eastern (*A. pellucida*; red), southern (*A. meridiana*; green), scaly (*A. vivax*; yellow), naked (*A. beanii*; purple) and Florida (*A. bifascia*; orange) sand darters using polygon data from Page  and  Burr  (2011) with approximate sampling locations for each species. The black dots in the red polygon denote the southern and northern populations of eastern sand darter. Naked and southern sand darters were sampled at the same site (black dot on green and purple overlap).

The *Ammocrypta* genus (Percidae) is a small monophyletic clade that is well suited for examining intra-and inter-specific differences in thermal tolerance. There are six species within the clade: the western sand darter (*Ammocrypta clara)*, eastern sand darter (*Ammocrypta pellucida)*, southern sand darter (*Ammocrypta meridiana)*, scaly sand darter (*Ammocrypta vivax)*, naked sand darter (*Ammocrypta beanii)* and Florida sand darter (*Ammocrypta bifascia;*  Fig. 1; Near  *et al.*, 2011). The *Ammocrypta* species are ecologically similar benthic fishes, preferring sand substrate and demonstrating fossorial behaviour (i.e. burying in the sand). The clade demonstrates high niche conservatism among all but the eastern sand darter (Mcnyset, 2009) and is distributed across a large latitudinal gradient within North America (Fig. 2). The approximate latitudinal ranges of Florida sand darter (29.7–31.7°N), southern sand darter (31.1–34.7°N), naked sand darter (30.3–35.8° N), scaly sand darter (29.7–37.1°N) and western sand darter (29.7–43.5°N) all have overlap, while only the latitudinal range of scaly and western sand darter overlap with eastern sand darter (36.5–46.7°N). Western sand darter has the largest latitudinal range and is listed as at-risk across the entire range (NatureServe, 2014). The eastern sand darter is the most northern of the species and occupies the second largest latitudinal range (36–46°N). Furthermore, the southern and northern populations of the eastern sand darter are genetically distinct, with the northern population listed as Threatened under the Canadian *Species at Risk Act* while the most southern population is not considered at-risk (Ginson  *et al.*, 2015; COSEWIC, 2022). The scaly and naked sand darters, and the southern and naked sand darters, are sympatric and syntopic (can be captured together in the same site and in the same habitat). Sand darter species are also morphologically similar in appearance, except the naked and Florida sand darters, which are sister taxa that have reduced scales only found along the lateral line and on the caudal peduncle (Page  and  Burr, 2011).

The aim of this study was to assess the extent of intra- and inter-specific differences in the upper thermal limits in *Ammocrypta*. Specifically, we assessed among-population (i.e. intra-specific) differences in the critical thermal maximum and agitation temperature of eastern sand darter, as the species has one of the largest latitudinal ranges in the genus. In addition, we assessed within-genus differences (i.e. inter-specific) in the critical thermal maximum and agitation temperature among five of the six *Ammocrypta* species.

## Materials and methods

### Field sampling

Thermal tolerance of the eastern sand darter has been assessed in the northern part of the species’ range by Firth  *et al.*  (2021), and the same methods and 2019 data were used in this study for comparability. Briefly, the northern population of eastern sand darter was sampled monthly in the Grand River, Ontario, Canada from June–November 2019. For seasonal comparability, only the June–August data were used in this study. All other *Ammocrypta* spp. field sampling took place in the USA from April–August 2023 (Fig. 2). The southern population of eastern sand darter was sampled in the Red River, Kentucky (April, May, August). Naked sand darter was sampled in Oakmulgee Creek (April) and the Cahaba River, Alabama (April, June) and southern sand darter was sampled in the Cahaba River, Alabama (April, June). Florida sand darter was sampled in the Yellow River, Alabama (May), and scaly sand darter was sampled in the Strong River and Oakatoma Creek, Mississippi (May). Due to low catchability, some species were sampled at multiple sites within the same week. For specific details on sampling sites and dates, see Table S1.

*Ammocrypta* spp. were captured in the sand-dominated depositional zones of the waterways using a straight seine net (1.8 × 3.0 m, 3.2 mm mesh) or bag seine (1.8 × 6.0 m, 3.2 mm mesh). Once captured, fish were held in a 5-gallon (18.9 L) bucket with an aerator and continuous water temperature monitoring to maintain field-acclimatized temperature for 30 minutes to 4 hours until testing. Western sand darter was not included in the study as only three specimens were caught during field sampling.

### Habitat measurements

Following Firth  *et al.*  (2021), water temperature (°C), dissolved oxygen (mg/L), conductivity ($\mathrm{\mu}$S/cm) and turbidity (NTU) were measured at the mid-point of each capture site at the bottom of the water column after fish collection once sediment was given time to settle using a YSI ProDSS Multiparameter digital water quality meter (Table 1).

### Upper thermal limits

All critical thermal maximum trials were conducted streamside following the protocol described in Firth  *et al.*  (2021) for comparability. Trials were started at the temperature that the fish were acclimated to in the wild at the time of capture. Trial water was obtained directly from the river, so each sampling day has a different acclimatization temperature based on the thermal regime of each river. Acclimatization temperature was calculated as the average of the start temperatures for each trial run in a day. For each trial, a single fish was placed in a mesh breeder box (16.5 × 12.5 × 13 cm) within a 37.8 L glass aquarium (51 × 25.5 × 30.5 cm; average ratio of fish mass to water volume is 1:38) and left to settle for 5 minutes. After the 5-minute settling period, temperature was heated by 0.3°C per minute (0.31 ± 0.001) using a Julabo portable immersion circulator (Julabo, Seelbach, Germany; Fig. S1). Temperature was increased until the fish demonstrated loss of equilibrium, denoted as the CT_max_ value. After loss of equilibrium, each fish was placed in a recovery bin with mixed (~50:50) river and trial water. After recovery (30–60 minutes), the total length (mm) and mass (g) of each fish were measured. The fish were then tagged with a visible implant elastomer tag (Northwest Marine Technology, Inc.) to avoid re-using individuals later in subsequent trials. All trials were recorded using a GoPro HERO4 Black model, and videos were analysed to score agitation temperature and to confirm the accuracy of the loss of equilibrium determinations. B. Firth blindly scored all videos for consistency and to reduce observer bias. Agitation temperature was scored as erratic swimming and digging behaviour towards the sides of the tank. The health status (e.g. parasite load) of all fish was unknown. Between trials, the aquarium was fully drained and refilled with fresh river water.

**Table 1:** Sample size and mean ± standard error fish mass (g), total length (mm), acclimatization temperature (°C), dissolved oxygen (mg/L), turbidity (NTU) and conductivity ($\mu$S/cm) for two populations of eastern sand darter (*A. pellucida*; N = Northern, S = Southern) and naked (*A. beanii*), southern (*A. meridiana*), Florida (*A. bifascia*) and scaly (*A. vivax*) sand darters at each sample time

**Species**	**Sample time**	**Sample** s**ize (*n*)**	**Mass (g)**	**Length (mm)**	**Temperature (°C)**	**Dissolved oxygen (mg/L)**	**Turbidity (NTU)**	**Conductivity (** $\boldsymbol{\mathrm{\mu}}$ **S/cm)**
N. Eastern	June	26	1.35 ± 0.08	62.42 ± 1.02	17.11 ± 0.15	8.88 ± 0.04	5.52 ± 0.30	639.33 ± 0.36
	July	25	1.25 ± 0.06	59.68 ± 0.87	25.02 ± 0.17	6.75 ± 0.11	7.64 ± 1.06	807.76 ± 8.91
	August	27	1.42 ± 0.05	63.00 ± 0.69	24.09 ± 0.19	6.95 ± 0.14	7.19 ± 0.22	831.18 ± 0.36
S. Eastern	April	19	0.83 ± 0.07	53.45 ± 0.50	14.00 ± 0.26	9.86 ± 0.07	3.05 ± 0.07	97.97 ± 0.19
	May	16	1.10 ± 0.06	53.63 ± 0.93	20.33 ± 0.21	8.59 ± 0.09	0.96 ± 0.01	196.30 ± 1.03
	August	26	1.21 ± 0.03	56.85 ± 0.37	23.71 ± 0.18	7.84 ± 0.07	6.88 ± 0.00	159.81 ± 0.29
Naked	April	16	0.55 ± 0.06	46.88 ± 1.45	18.74 ± 0.32	8.92 ± 0.04	11.61 ± 0.39	103.76 ± 24.38
	June	14	0.84 ± 0.05	51.57 ± 1.28	26.97 ± 0.12	8.04 ± 0.00	3.08 ± 0.00	289.50 ± 0.00
Southern	April	14	1.05 ± 0.05	56.93 ± 0.67	20.47 ± 0.21	8.53 ± 0.01	7.39 ± 0.16	200.10 ± 1.05
	June	11	1.33 ± 0.07	58.00 ± 0.76	26.94 ± 0.22	7.33 ± 0.00	5.33 ± 0.00	269.20 ± 0.00
Florida	May	18	1.25 ± 0.09	58.27 ± 1.13	21.92 ± 0.07	7.31 ± 0.00	2.23 ± 0.00	118.70 ± 0.00
Scaly	May	17	1.26 ± 0.12	57.12 ± 1.99	22.40 ± 0.16	6.90 ± 0.10	35.18 ± 1.78	47.43 ± 2.72

Data were averaged from each sampling event (i.e. 1–4 day sampling period).

### Statistical analysis

A linear mixed effects model with a Gaussian distribution was used to assess the fixed effects of acclimatization temperature, eastern sand darter population, and mass on the dependent variables (CT_max_ and T_ag_). Mass was included as a fixed predictor variable as it was previously shown to impact CT_max_ in eastern sand darter (Firth  *et al.*, 2021). Sampling event, the group of days where individuals were assessed within a similar temperature range, was included as a random effect. The models were assessed using the R package lme4 (Bates  *et al.*, 2015) with population mean and slope (i.e. ARR) contrasts assessed using ‘emmeans’ and ‘emtrends’ from the R package ‘emmeans’(Lenth, 2016) at 23.5°C where data for northern and southern eastern sand darter overlapped.

**Table 2:** Beta (*β*) estimate, standard error (SE), degrees of freedom (df), *t*-value (*t*) and *P*-values (*P*) for the equally plausible candidate linear mixed effect models for testing of significant differences in CT_max_ and T_ag_ among eastern sand darter (*A. pellucida*) populations

**Model**	**Variable**	$\boldsymbol{\beta}$	**SE**	**df**	***t***	***P***
**CT**_**max**_ **models**						
Temp + (1|SampleEvent)	**Temp**	**0.53**	**0.04**	**2.33**	**12.45**	**.003**
Temp × mass +	**Temp**	**0.44**	**0.07**	**26.44**	**6.49**	**.000**
(1|SampleEvent)	Mass	−1.15	1.07	121.54	−1.08	.28
	Temp × Mass	0.07	0.05	125.85	1.37	.17
Temp + mass +	**Temp**	**0.52**	**0.04**	**2.60**	**11.72**	**.003**
(1|SampleEvent)	Mass	0.29	0.21	136.00	1.35	.18
Temp + population +	**Temp**	**0.24**	**0.11**	**9.53**	**2.25**	**.05**
(1|SampleEvent)	Population (red)	−0.95	1.37	1.36	−0.69	.59
**T**_**ag**_ **models**						
Temp × population +	**Temp**	**0.82**	**0.13**	**2.37**	**6.32**	**.02**
(1|SampleEvent)	Population (red)	8.40	3.81	2.38	2.20	.14
	Temp × population (red)	−0.37	0.18	2.36	−2.08	.15
Temp × population + mass +	**Temp**	**0.80**	**0.14**	**2.25**	**5.76**	**.02**
(1|SampleEvent)	Population (red)	7.76	4.11	2.23	1.89	.19
	Mass	−0.54	0.40	120.14	−1.35	.18
	Temp × population (red)	−0.35	0.19	2.20	−1.82	.20

Bolded values indicate significance at *α* = 0.05.

**Figure 3:**
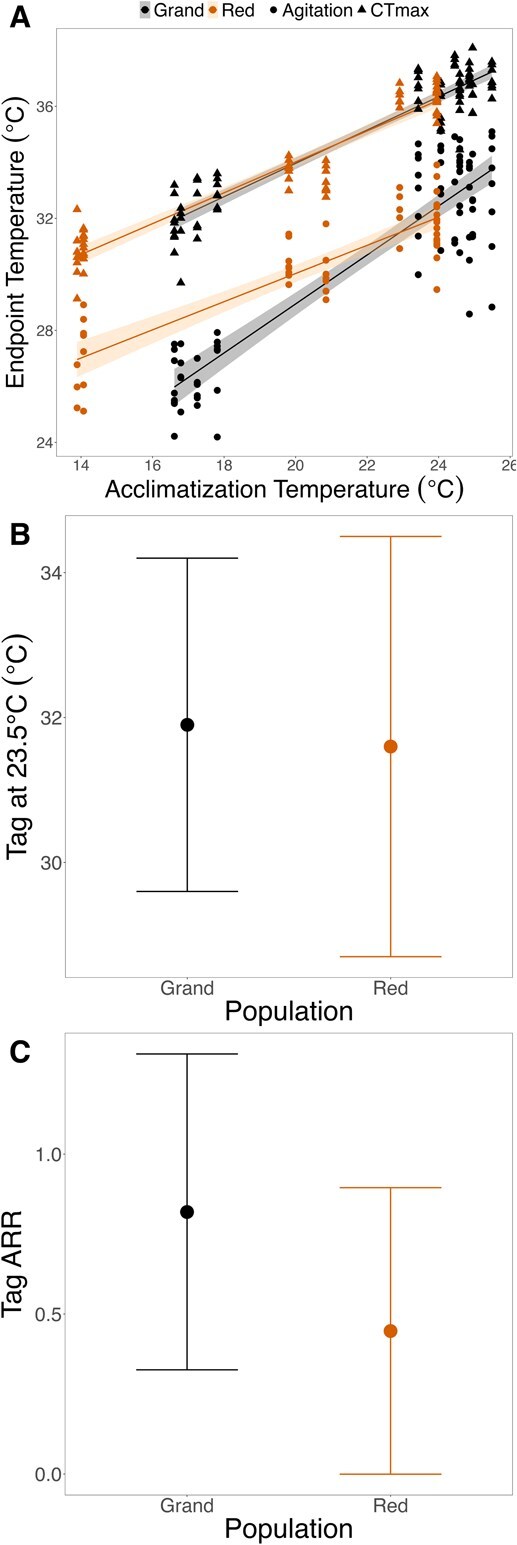
Intra-specific differences in critical thermal maximum (triangles) and agitation temperature (circles) between southern (orange) and northern (black) populations of eastern sand darter (*A. pellucida*) across acclimatization temperature (average temperature per day; **A**). Agitation temperature mean values (**B**) and agitation temperature estimated ARR (**C**) from linear mixed effect T_ag_ model 1 at 23.5C for both populations. Shaded areas (A) and error bars (B and C) represent the 95% confidence intervals.

A second linear mixed effects model with acclimatization temperature and mass as fixed effects and species and sampling event as random effects was conducted to assess both dependent variables (i.e. CT_max_ and T_ag_). Species slopes were allowed to vary with acclimatization temperature. Sampling event, the group of days where individuals were assessed at the same site within a similar temperature range, was included as a random effect nested within species (i.e. each species-specific sampling event was allowed to have a random intercept). A linear mixed effects model with a Gaussian distribution was also used to assess the fixed effects of acclimatization temperature, *Ammocrypta* species, and mass on the dependent variables (CT_max_ and T_ag_). In the among-species analysis, the northern and southern populations of eastern sand darter were combined. Given the reduced temporal range for which data were available for all species, regression analysis was conducted with species differences assessed with temperature set to 21.91°C. The temperature represents the average Florida (21.91 ± 0.07) sand darter acclimatization temperature for the single day of sampling for this species, which avoids statistical issues associated with extrapolation outside the range of data for which regression analysis was run (Zar, 2018). The models were assessed using the R package lme4 (Bates  *et al.*, 2015). Species mean and slope (i.e. ARR) contrasts were assessed using ‘emmeans’ and ‘emtrends’, respectively, with Tukey adjustment from the R package ‘emmeans’ (Lenth, 2016).

For both intra- and inter-specific comparisons, the Akaike Information Criterion adjusted for small samples (AIC_c_; Symonds  and  Moussalli, 2011) was used for model selection to evaluate support for all main effects and their single interaction combinations. Models assessed always included temperature due to temperature being a known key predictor of CT_max_. Only one interaction term was included in the models as multiple interactions with a variable introduced covariation in the model. The temperature × species interaction term was included since species have been shown to have different acclimation capacity (i.e. slope) across temperatures (Comte  and  Olden, 2017*a*). The temperature × mass interaction term was included since mass increased over the seasons. However, it is important to note there was mass overlap between temperatures for each species. The species × mass interaction was included since not all species have demonstrated a change in CT_max_ with mass and the relationship is unknown for each of the tested species. Due to the weak correlation (*r* = 0.2, *P* > .001) between acclimatization temperature and mass, variance inflation factors for multicollinearity were tested for each model. Models with ΔAIC_c_ < 2 were considered as equally plausible (Burnham  and  Anderson, 2002). To compare models with different fixed effects in AIC_c_, restricted maximum likelihood was not incorporated and maximum likelihood was used (Faraway, 2006; Zuur  *et al.*, 2009). After finding the best models, restricted maximum likelihood was used to assess the models.

## Results

### Eastern sand darter intra-specific comparison

Four models yielded ΔAIC_c_ < 2 for eastern sand darter intra-specific CT_max_ model selection (Table S2). The best-fit model included only acclimatization temperature (*w*_i_ = 0.27). The second model included acclimatization temperature and the interaction with mass (ΔAIC_c_ = 0.31, *w*_i_ = 0.23). The third model included acclimatization temperature and mass (ΔAIC_c_ = 0.49, *w*_i_ = 0.21), and the fourth model included acclimatization temperature and population (ΔAIC_c_ = 1.99, *w*_i_ = 0.10). The random effect of sample event in all four candidate models explained 25.08, 20.86, 27.01 and 85.14% of the remaining variation. In all four candidate models, CT_max_ was positively correlated with acclimatization temperature (*β* = 0.53, 0.44, 0.52 and 0.24, respectively; Table 2, Fig. 3A), and there were no other significant effects (Table 2). In the fourth candidate model, CT_max_ was not significantly different between the northern (35.5 ± 0.96, 95% Cl 32.7–38.4°C) and southern (34.6 ± 1.08, 95% Cl 31.7–37.5°C) eastern sand darter populations at the acclimatization temperature of 23.5°C (*β* = 0.95, SE = 1.38, *t* = 0.68, *P* = .54; Fig. S2B).

Two models yielded ΔAIC_c_ < 2 for eastern sand darter intra-specific T_ag_ model selection (Table S3). The best-fit model included acclimatization temperature and the interaction with population (*w*_i_ = 0.37), and the second model included acclimatization temperature and the interaction with population plus mass (ΔAIC_c_ = 0.83, *w*_i_ = 0.24). The random effect of sample event was in both candidate models, representing 28.88 and 32.84% of the remaining variance. In both candidate models, T_ag_ was positively correlated with acclimatization temperature (*β* = 0.82 and 0.80, respectively; Table 2, Fig. 3A), and there were no other significant effects (Table 2). In the best candidate model, T_ag_ was not significantly different between the northern (31.9 ± 0.54, 95% Cl 29.6–34.2°C) and southern (31.6 ± 0.73, 95% Cl 28.7–34.5°C) eastern sand darter populations at the acclimatization temperature of 23.5°C (*β* = 0.35, SE = 0.91, *t* = 0.38, *P* = .74; Fig. 3B). Further, the T_ag_ slope was not significantly different between the northern (0.82 ± 0.15, 95% Cl 0.33–1.31°C) and southern (0.45 ± 0.13, 95% Cl −0.0005-0.89°C) eastern sand darter populations at the acclimatization temperature of 23.5°C (*β* = 0.37, SE = 0.20, *t* = 1.89, *P* = .16; Fig. 3C) in the best candidate model. Similarly, model 2 demonstrated no significant results for the population contrasts (Fig. S2A and C).

**Table 3:** Beta (*β*) estimate, standard error (SE), degrees of freedom (df), *t*-value (*t*) and *P*-values (*P*) for the equally plausible candidate linear mixed effect models for testing of significant differences in CT_max_ and T_ag_ between sand darter species (eastern *A. pellucida*; naked *A. beanie*; southern *A. meridiana*; Florida *A. bifascia*; and scaly *A. vivax*)

**Model**	**Variable**	$\boldsymbol{\beta}$	**SE**	**df**	***t***	***P***
**CT**_**max**_ **models**						
Temp × Species + Mass +	**Temp**	**0.52**	**0.04**	**3.79**	**13.01**	**.0003**
(1|Species:SampleEvent)	**Mass**	**0.37**	**0.17**	**218.79**	**2.14**	**.03**
	**Species (Florida)**	**−1.53**	**0.45**	**3.29**	**−3,38**	**.04**
	**Species (Naked)**	**−1.31**	**0.32**	**4.66**	**−4.02**	**.01**
	Species (Scaly)	−1.09	0.57	4,02	−1.89	.13
	Species (Southern)	0.32	0.39	3.58	0.82	.46
	Temp × Naked	0.007	0.08	3.89	0.09	.94
	Temp × Scaly	0.37	0.71	5.73	0.52	.62
	Temp × Southern	−0.18	0.10	3.91	−1.81	.15
Temp × Species	**Temp**	**0.57**	**0.01**		**35.79**	**.00**
	**Species (Florida)**	**−1.59**	**0.19**		**−8.44**	**.00**
	**Species (Naked)**	**−1.57**	**0.15**		**−10.27**	**.00**
	**Species (Scaly)**	**−0.98**	**0.28**		**−3.48**	**.00**
	Species (Southern)	0.18	0.18		1.00	.32
	Temp × Naked	−0.03	0.03		−0.73	.47
	Temp × Scaly	0.003	0.42		0.007	.99
	**Temp × Southern**	**−0.20**	**0.05**		**−4.05**	**.00**
Temp × Species + Mass	**Temp**	**0.56**	**0.02**		**34.20**	**.00**
	Mass	0.24	0.17		1.45	.15
	**Species (Florida)**	**−1.59**	**0.19**		**−8.47**	**.00**
	**Species (Naked)**	**−1.43**	**0.18**		**−7.90**	**.00**
	**Species (Scaly)**	**−1.09**	**0.29**		**−3.75**	**.00**
	Species (Southern)	0.21	0.18		1.18	.24
	Temp × Naked	−0.03	0.04		−0.81	.42
	Temp × Scaly	0.22	0.45		0.49	.62
	**Temp × Southern**	**−0.20**	**0.05**		**−4.14**	**.00**
Temp × Species +	**Temp**	**0.54**	**0.04**	**3.53**	**14.34**	**.00**
(1|Species:SampleEvent)	**Species (Florida)**	**−1.53**	**0.42**	**3.26**	**−3.62**	**.03**
	**Species (Naked)**	**−1.52**	**0.29**	**3.94**	**−5.21**	**.007**
	Species (Scaly)	−0.92	0.53	3.95	−1.73	.16
	Species (Southern)	0.26	0.36	3.58	0.72	.51
	Temp × Naked	0.006	0.07	3.91	0.09	.93
	Temp × Scaly	0.04	0.66	5.55	0.06	.96
	Temp × Southern	−0.18	0.09	3.94	−1.87	.14

(Continued)

**Table 3:** Continued.

**Model**	**Variable**	$\boldsymbol{\beta}$	**SE**	**df**	***t***	***P***
Temp + Species + Mass +	**Temp**	**0.50**	**0.03**	**7.55**	**14.59**	**.00**
(1|Species:SampleEvent)	**Mass**	**0.35**	**0.17**	**217.70**	**2.05**	**.04**
	**Species (Florida)**	**−1.50**	**0.48**	**6.19**	**−3.11**	**.02**
	**Species (Naked)**	**−1.30**	**0.34**	**8.45**	**−3.79**	**.005**
	Species (Scaly)	−0.81	0.40	7.93	−2.05	.07
	Species (Southern)	0.07	0.39	6.74	0.17	.87
**T**_**ag**_ **models**						
Temp + (1|Species:SampleEvent)	**Temp**	**0.57**	**0.07**	**13.11**	**7.83**	**.00**
Temp + Mass +	**Temp**	**0.57**	**0.07**	**13.33**	**7.87**	**.00**
(1|Species:SampleEvent)	Mass	−0.45	0.34	174.64	−1.34	.18
Temp × Mass +	**Temp**	**0.69**	**0.13**	**77.44**	**5.35**	**.00**
(1|Species:SampleEvent)	Mass	−0.59	0.36	179.54	−1.65	.10
	Temp × Mass	−0.11	0.10	187.34	−1.16	.25

Bolded values indicate significance at *α* = 0.05.

**Figure 4:**
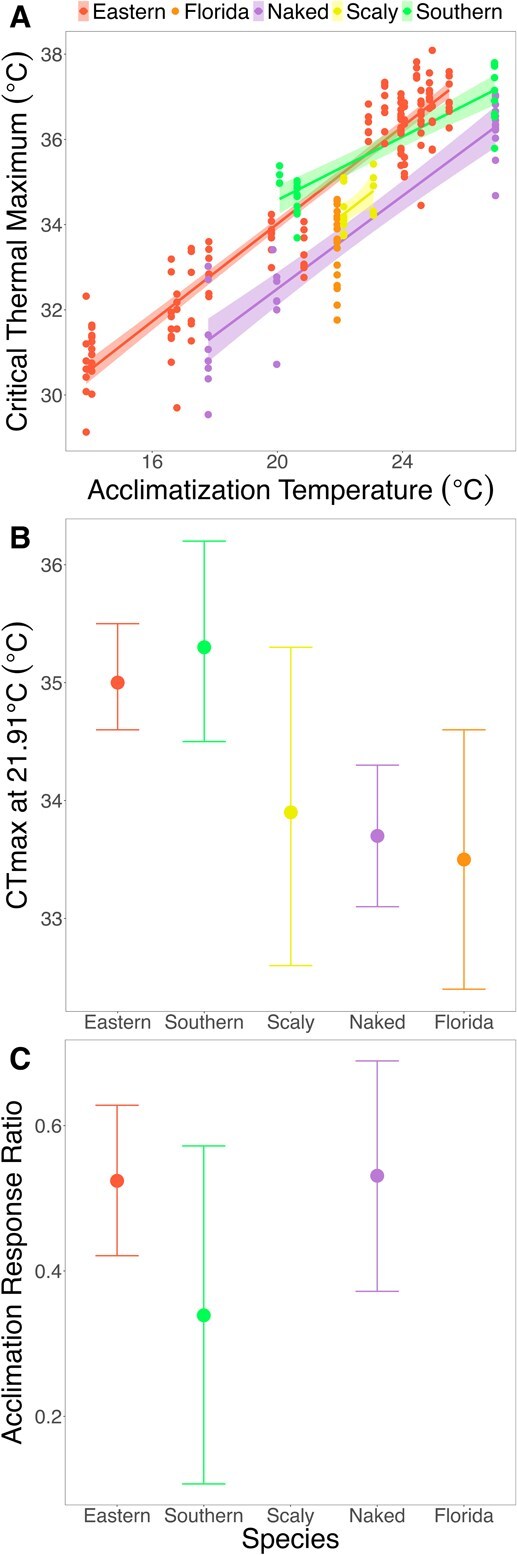
Inter-specific differences in critical thermal maximum (**A**) for eastern (*A. pellucida*; red), southern (*A. meridiana*; green), scaly (*A. vivax*; yellow), naked (*A. beanii*; purple) and Florida (*A. bifascia*; orange) sand darters across acclimatization temperatures and the critical thermal maximum mean values (**B**) and estimated ARR(**C**) from linear mixed effect model 1 at 21.91 °C for each species. Shaded areas (A) and error bars (B and C) represent the 95% confidence intervals.

### Species inter-specific comparison

In the CT_max_ model with species as a random effect, CT_max_ was positively correlated with acclimatization temperature (*β* = 0.46, SE = 0.06, *t* = 7.95, *P* = .00) and mass (*β* = 0.43, SE = 0.17, *t* = 2.46, *P* = .01), with larger fish having a greater CT_max_. The interaction between mass and temperature was not significant (*β* = 0.06, SE = 0.04, *t* = 1.25, *P* = .21). Sampling event explained 14.92% of the remaining variance while species and the variation of the species slopes explained 33.69 and 0.1% of the remaining variance, respectively. For the AIC_c_ models, five models yielded ΔAIC_c_ < 2 for species inter-specific CT_max_ model selection (Table S4). The best-fit model included acclimatization temperature and the interaction with species plus mass (*w*_i_ = 0.27). The second model included acclimatization temperature and the interaction with species (ΔAIC_c_ = 1.06, *w*_i_ = 0.16). The third candidate model included acclimatization temperature and the interaction with species plus mass (ΔAIC_c_ = 1.09, *w*_i_ = 0.16). The fourth candidate model included acclimatization temperature and the interaction with species (ΔAIC_c_ = 1.34, *w*_i_ = 0.14), and the fifth candidate model included acclimatization temperature, species and mass (ΔAIC_c_ = 1.43, *w*_i_ = 0.13). The random effect of sample event was only included in the first, fourth and fifth models, explaining 24.72, 19.10 and 25.21% of the remaining variance, respectively. In all models, CT_max_ was positively correlated with acclimatization temperature (*β* = 0.52, 0.57, 0.56, 0.54 and 0.50, respectively; Table 3, Fig. 4A). In the best fit model, CT_max_ was positively correlated with mass (*β* = 0.37, SE = 0.17, df = 218.79, *t* = 2.14, *P* = .03), with larger fish having a greater CT_max_ (Table 3). Mass was also significantly positively correlated with CT_max_ in the fifth candidate model, but not the third (Table 3). In the best fit model, eastern sand darter had a significantly higher CT_max_ at 21.91°C than Florida sand darter (*β* = −1.53, *P* = .04) and naked sand darter (*β* = −1.31, *P* = .01), but did not differ significantly from scaly sand darter (*β* = −1.09, *P* = .13) or southern sand darter (*β* = 0.32, *P* = .46; Fig. 4B). However, the more conservative Tukey’s *post hoc* pairwise comparisons of CT_max_ at 21.91°C for the best fit model indicated that eastern sand darter (35.0 ± 0.18, 95% Cl 34.6–35.3°C) was only significantly different from naked sand darter (33.7 ± 0.27, 95% Cl 33.1–34.3°C; *P* = .03) and not Florida (33.5 ± 0.42, 95% Cl 32.4–34.6°C; *P* = .09), scaly (33.9 ± 0.55, 95% Cl 32.6–35.3°C; *P* = .41) or southern sand darter (35.3 ± 0.35, 95% Cl 34.5–36.2°C; *P* = .91). There were no other significant pairwise comparisons between all the species in the best candidate model (*P*$\ge$ .05; Table 4). In the best fit model, CT_max_ slope (i.e. ARR) did not significantly differ between eastern sand darter, naked sand darter (*β* = 0.007, *P* = .94), scaly sand darter (*β* = 0.37, *P* = .62) or southern sand darter (*β* = −0.18, *P* = .15; Fig. 4C) and there were no significant pairwise comparisons (Table 5).

In all five candidate models, Florida and naked sand darter CT_max_ were significantly different from eastern sand darter. In the second and third candidate models, scaly sand darter CT_max_ and southern sand darter slope (i.e. ARR) were significantly different from eastern sand darter (Table 3). Pairwise comparisons for CT_max_ mean and slope of all five of the candidate models are provided in Table 4 and 5 (Fig. S3).

**Figure 5:**
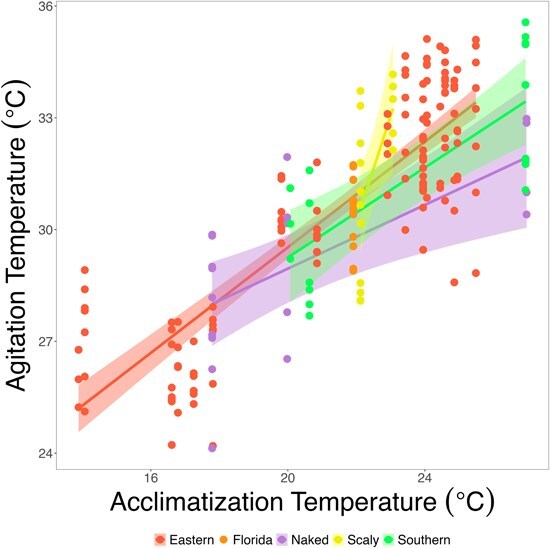
Inter-specific differences in agitation temperature for eastern (*A. pellucida*; red), southern (*A. meridiana*; green), scaly (*A. vivax*; yellow), naked (*A. beanii*; purple) and Florida (*A. bifascia*; orange) sand darters across acclimatization temperatures. Shaded areas represent the 95% confidence intervals.

In the T_ag_ model with species as a random effect, T_ag_ was positively correlated with acclimatization temperature (*β* = 0.69, SE = 0.12, *t* = 5.53, *P* = .00). Mass (*β* = −0.60, SE = 0.35, *t* = −1.70, *P* = .09) and the interaction between mass and temperature (*β* = −0.10, SE = 0.10, *t* = −1.08, *P* = .28) was not significant. Sampling event explained 25.13% of the remaining variance while species and the variation of the species slopes explained 1.99 and 0.04% of the remaining variance, respectively. For the AIC_c_ models, three models yielded ΔAIC_c_ < 2 for species inter-specific T_ag_ model selection (Table S5). The best-fit model included acclimatization temperature (*w*_i_ = 0.34). The second model included acclimatization temperature and mass (ΔAIC_c_ = 0.29, *w*_i_ = 0.30), and the third candidate model included acclimatization temperature and the interaction with mass (ΔAIC_c_ = 1.26, *w*_i_ = 0.18). The random effect of sample event was in all three models, representing 28.63, 29.08 and 31.71% of the remaining variance, respectively. In all models, T_ag_ was positively correlated with acclimatization temperature (*β* = 0.57, 0.57 and 0.69, respectively; Table 3, Fig. 5). There were no other significant effects in any of the models (Table 3).

## Discussion

By assessing upper thermal limits within and among species in *Ammocrypta*, this study provides insight into the importance of understanding variation in the thermal response of freshwater fishes for informing conservation decisions. The study demonstrated no intra-specific differences in CT_max_ or T_ag_ between genetically distinct northern and southern populations of eastern sand darter. Although the population model term was not significant, populations were important enough to be in both T_ag_ candidate models. The study also demonstrated minimal inter-specific differences in CT_max_ and no difference in T_ag_ within *Ammocrypta*. The CT_max_ differences between species depended on the candidate model; however, eastern sand darter always had higher thermal tolerance compared to naked sand darter. Furthermore, the study demonstrates the significant effect of mass on CT_max_ with larger fish having a larger CT_max_. Overall, this study underlines the importance of assessing intra- and inter-specific differences in near-lethal (i.e. CT_max_) and non-lethal (i.e. T_ag_) metrics for a better understanding of species’ acclimation capacity for future environmental change.

### Eastern sand darter intra-specific comparison

There were no intra-specific differences in CT_max_ between eastern sand darter populations despite their established genetic divergence. Similarly, intra-specific differences in CT_max_ were not observed in genetically divergent lake trout *(Salvelinus namaycush*; Kelly  *et al.*, 2014) or redband trout (*Oncorhynchus mykiss*; Rodnick  *et al.*, 2004) populations. Nevertheless, many species have demonstrated intra-specific differences in CT_max_ (e.g. mummichog: *Fundulus heteroclitus*; Fangue  *et al.*, 2006). Populations in warmer habitats typically have greater thermal tolerance, which has been attributed to plasticity and/or local adaptation to differences in thermal regimes (Fangue  *et al.*, 2006; Anttila  *et al.*, 2013; McKenzie  *et al.*, 2021; Zillig  *et al.*, 2023). The average daily difference in water temperature between the Grand and Red rivers (~0.89°C difference July–August 2019), if consistent across time, may not have been large enough to have induced local adaptation in CT_max_. Although genetic divergence has been observed between eastern sand darter populations (Ginson  *et al.*, 2015), such divergence could have been elicited in response to local adaptation to other habitat variables (e.g. oxygen) and/or non-selective genetic drift due to lack of gene flow.

**Table 4:** Beta (*β*) estimate, standard error (SE), *t*-value (*t*) and *P*-value (*P*) from Tukey’s HSD *post hoc* test results for testing mean pairwise significant sand darter species (eastern *A. pellucida*; naked *A. beanie*; southern *A. meridiana*; Florida *A. bifascia*; and scaly *A. vivax*) differences for the five equally plausible candidate CT_max_ models

**Model**		**Eastern–Florida**	**Eastern–Naked**	**Eastern–Scaly**	**Eastern–Southern**	**Florida–Naked**	**Florida–Scaly**	**Florida–Southern**	**Naked–Scaly**	**Naked–Southern**	**Scaly–Southern**
Temp × Species + Mass + (1|SampleEvent)	***β***	1.53	**1.31**	1.09	−0.32	−0.22	−0.45	−1.85	−0.23	−1.63	−1.40
	**SE**	0.45	**0.33**	0.58	0.39	0.50	0.69	0.54	0.63	0.44	0.65
	***t***	3.38	**4.02**	1.89	−0.82	−0.44	−0.65	−3.41	−0.36	−3.72	−2.16
	***P***	.09	**.03**	.41	.91	.99	.96	.09	.99	.05	.31
Temp × Species	***β***	**1.59**	**1.57**	**0.98**	−0.18	−0.02	−0.61	**−1.77**	−0.59	**−1.75**	**−1.16**
	**SE**	**0.19**	**0.15**	**0.28**	0.18	0.22	0.33	**0.24**	0.31	**0.22**	**0.32**
	***t***	**8.44**	**10.27**	**3.48**	−1.00	−0.08	−1.86	**−7.33**	−1.92	**−8.15**	**−3.63**
	***P***	**.00**	**.00**	**.005**	.85	1.00	.34	**.00**	.31	**.00**	**.003**
Temp × Species + Mass	***β***	**1.59**	**1.43**	**1.09**	−0.21	−0.16	−0.50	**−1.80**	−0.34	**−1.64**	**−1.30**
	**SE**	**0.19**	**0.18**	**0.29**	0.18	0.25	0.33	**0.24**	0.35	**0.23**	**0.33**
	***t***	**8.47**	**7.90**	**3.75**	−1.18	−0.65	−1.50	**−7.46**	−0.97	**−7.23**	**−3.90**
	***P***	**.00**	**.00**	**.002**	.76	.97	.56	**.00**	.87	**.00**	**.001**
Temp × Species + (1|SampleEvent)	***β***	1.53	**1.52**	0.92	−0.26	−0.006	−0.61	−1.79	−0.60	**−1.79**	−1.18
	**SE**	0.43	**0.29**	0.54	0.36	0.46	0.64	−0.51	0.57	**0.41**	0.60
	***t***	3.62	**5.2**	1.73	−0.72	−0.01	−0.95	−3.53	−1.07	**−4.40**	−1.96
	***P***	.07	**.01**	.49	.94	1.00	.87	.08	.82	**.03**	.38
Temp + Species + Mass + (1|SampleEvent)	***β***	1.50	**1.30**	0.81	−0.07	−0.20	−0.69	−1.57	−0.49	−1.37	−0.88
	**SE**	0.48	**0.34**	0.40	0.39	0.54	0.57	0.56	0.45	0.44	0.48
	***t***	3.11	**3.78**	2.50	−0.17	−0.38	−1.22	−2.81	−1.09	−3.12	−1.84
	***P***	.09	**.02**	.32	1.00	.99	.74	.12	.81	.07	.41

Bolded values indicate significance at *α* = 0.05.

**Table 5:** Beta (*β*) estimate, standard error (SE), *t*-value (*t*) and *P*-value (*P*) from Tukey’s HSD *post hoc* test results for testing slope pairwise significant sand darter species (eastern *A. pellucida*; naked *A. beanie*; southern *A. meridiana*; Florida *A. bifascia*; and scaly *A. vivax*) differences for the equally plausible candidate CT_max_ models (only the top four contained temperature species interactions)

**Model**		**Eastern–Naked**	**Eastern–Southern**	**Naked–Southern**
Temp × Species + Mass + (1|SampleEvent)	***β***	−0.006	0.18	0.19
	**SE**	0.08	0.10	0.11
	***t***	−0.09	1.79	1.67
	***P***	.99	.36	.41
Temp × Species	***β***	0.03	**0.20**	**0.17**
	**SE**	0.04	**0.05**	**0.06**
	***t***	0.73	**4.05**	**3.04**
	***P***	.88	**.00**	**.01**
Temp × Species + Mass	***β***	0.03	**0.20**	**0.17**
	**SE**	0.04	**0.05**	**0.06**
	***t***	0.81	**4.13**	**3.07**
	***P***	.85	**.00**	**.01**
Temp × Species + (1|SampleEvent)	***β***	−0.006	0.18	0.19
	**SE**	0.07	0.10	0.11
	***t***	−0.09	1.86	1.73
	***P***	.99	.34	.38

Florida and scaly sand darter are not included in the pairwise comparisons due to only having temperature sampling events. Bolded values indicate significance at α = 0.05.

CT_max_ significantly increased with acclimatization temperature with eastern sand darter demonstrating a higher degree of plasticity in CT_max_ across seasonal temperatures (i.e. average ARR of 0.43 from the top four models) compared to other mid-latitude fishes (~0.35; Morley  *et al.*, 2019) and widely distributed freshwater fishes (0.37; Comte  and  Olden, 2017*a*; see Firth  *et al.*, 2021 for discussion). A high degree of seasonal plasticity and the short generation time of eastern sand darter can result in longer temporal buffers for evolutionary rescue to occur before loss of thermal safety (Bell, 2013; Morley  *et al.*, 2019). However, a high ARR is limited, as CT_max_ is suggested to have a hard upper limit (i.e. ‘concrete ceiling’) due to physiological constraints, and CT_max_ plasticity has been shown to decrease as acclimation temperature increases (Gunderson  and  Stillman, 2015; Sandblom  *et al.*, 2016). Further, zebrafish with a short generation time demonstrated low potential for evolutionary rescue with CT_max_ only increasing by 0.22°C after six generations of selecting for the highest CT_max_ (Morgan  *et al.*, 2020). Nevertheless, eastern sand darter could still have potential for limited evolutionary rescue in the short-term since temperate species typically have greater thermal safety margins than tropical species (Sunday  *et al.*, 2011; Rummer  *et al.*, 2014).

In addition to no intra-specific differences in CT_max_, eastern sand darter populations showed no difference in CT_max_ plasticity (i.e. ARR), with the interaction between population and acclimatization temperature absent in the top models. The lack of intra-specific differences in CT_max_ and CT_max_ plasticity across acclimatization temperature aligns with Kelly  *et al.*  (2014), who noted no differences in CT_max_ and the interaction between temperature and CT_max_ among tested lake trout populations. Absence of variation in CT_max_ and plasticity in CT_max_ between populations could imply increased vulnerability to future temperature increases due to the portfolio effect, which theorizes that greater variation can buffer the negative effects of environmental change, leading to greater chances of survival (Bolnick  *et al.*, 2011; McKenzie  *et al.*, 2021). For eastern sand darter, the lack of among-population variation in mean CT_max_ and CT_max_ plasticity reduces the genetic variation for selection to act upon, increasing the risk for negative consequences, including localized extirpation and extinction (e.g. Frankham, 2005; Rohr  *et al.*, 2018). However, selection can also act upon within-population variation that could buffer populations from local extirpation and potential extinction. Although eastern sand darter may have similar population CT_max_ and CT_max_ plasticity, implying increased vulnerability to environmental change, the degree of within- and among-population variation may still be sufficient for selection to act upon and provide a chance of evolutionary rescue.

Similar to CT_max_, we observed no differences in T_ag_ and T_ag_ plasticity (i.e. the interaction between population and acclimatization temperature) between the genetically distinct populations. Despite no population differences in T_ag_, population alone and its interaction with acclimatization temperature were included in both the top models, implying population is still important in predicting T_ag_. Wells  *et al.*  (2016) is the only other study to assess T_ag_ between populations but did not assess T_ag_ across a range of temperatures. Similar to our findings, they observed no differences in T_ag_ between brook trout (*Salvelinus fontinalis*) populations. Although T_ag_ plasticity was not significantly different between the populations, T_ag_ demonstrated high plasticity (slope = 0.63, average of northern, 0.82 and southern, 0.45 slopes) with acclimatization temperature. However, as temperatures increase, the agitation safety margin and agitation window (difference between CT_max_ and T_ag_) are shrinking since eastern sand darter plasticity in agitation (0.62) is not perfect (i.e. 1) and is greater than ARR for CT_max_ (0.43). Relatively little is known about the plasticity of T_ag_, and there is uncertainty about whether T_ag_ can continue to increase indefinitely until hitting CT_max_ or whether plasticity will begin to decrease at higher temperatures and plateau. Furthermore, it is unclear if sub-lethal thermal stress, such as peak heart rate and cellular stress (Bouyoucos  *et al.*, 2023), will continue to occur with T_ag_ across a range of temperatures.

### Species inter-specific comparison

Inter-specific differences in CT_max_ were minimal between *Ammocrypta* congeners (i.e. species within the genus). As a random effect, species explained a moderate amount (33.69%) of the remaining variance in CT_max_. Additionally, as a fixed effect, species was significant in every equally plausible candidate model thereby supporting the moderate importance of species in predicting CT_max_. Species-specific pairwise comparisons were not robust across all the top candidate models, but eastern sand darter always had a significantly higher CT_max_ than naked sand darter, implying some robust species differences. Observed species differences and the importance of species in predicting CT_max_ in our study need to be interpreted with caution, as our findings are limited due to unincorporated within- and among-site variation from *in situ* sampling. Field studies are beneficial by allowing for ecologically and biologically relevant findings due to the natural environment and prevailing abiotic and biotic variation. However, microhabitat temperature differences, unknown health- and feed-state (i.e. parasite load; digested or fasted) and varying lengths of recovery post-capture could contribute to within- and among-site variation leading to under- or overestimation of species effects. Another factor contributing to among-site variation is our use of single-timepoint temperature acclimatization, and we recognize that acclimation temperature and duration are important for predicting CT_max_ (Ruthsatz  *et al.*, 2024). For our study, we were unable to obtain long-term temperature measurements due to research timelines and the geographical logistics of long-distances logger deployment. Acclimation temperature is not instantaneous and is governed by the temporal thermal experience of the fish (thermal variability, season, duration; Morash  *et al.*, 2018; Turko  *et al.*, 2020; Stewart  *et al.*, 2023). Thus, our single-timepoint acclimation temperatures do not consider thermal variability between sites and previous seasonal differences in acclimation (i.e. prolonged exposure to warm temperatures in the summer could increase CT_max_), which could have contributed to over-or underestimates of species differences. However, other than for eastern sand darter, all data were collected in a single season (spring), and fish likely experienced similar seasonal thermal variability across the limited geographic range of sites sampled. Additionally, the acclimatization temperature that best predicted CT_max_ for three small-bodied fishes (pugnose shiner, blackchin shiner and redside dace [*Clinostomus elongatus*]) tested in the field, like *Ammocrypta,* was the mean temperature experienced by the fish only one and 2 days before testing. Thus, despite our lack of true acclimatization data, we believe the species is still of moderate importance for predicting CT_max_. To validate the importance of species on CT_max_, a lab study is needed to reduce the importance of environmental variation and other field-based complexity.

Understanding plasticity (i.e. ARR) in CT_max_ is important for interpreting species adaption and survival potential due to predicted future increases in water temperature. The *Ammocrypta* genus demonstrated seasonal plasticity in CT_max_ similar to other field-acclimatized species (Leclair  *et al.*, 2020; Turko  *et al.*, 2020). However, there were significant inter-specific differences in CT_max_ plasticity in two of the four models, with southern sand darter having a significantly lower ARR (0.36; average of all four models) compared to eastern (0.55) and naked sand darter (0.54). Nevertheless, southern sand darter ARR was still higher than the ARR of fantail, greenside and rainbow darter measured elsewhere (0.22, 0.25 and 0.31, respectively; Hlohowskyj  and  Wissing, 1985) and similar to the average among many freshwater fishes (0.37, Comte  and  Olden, 2017*b*; ~ 0.35 for mid-latitude species, Morley  *et al.*, 2019). Based on the ARR data, the southern sand darter has a lower acclimation capacity and, therefore, a decreased chance of evolutionary rescue compared to eastern and naked sand darter, but its ARR is still similar to the average of other freshwater fishes. In contrast, eastern and naked sand darter had higher ARRs, suggesting increased chances of evolutionary rescue. Thus, future increases in climate change could threaten southern sand darter more than the other *Ammocrypta* species.

In contrast to CT_max_, no species differences in T_ag_ or T_ag_ plasticity were observed, with species only explaining 2% of the remaining variance and not appearing in any of the candidate models. The only other study to assess T_ag_ between congeners found that T_ag_ was significantly different between pugnose and blackchin shiner, which aligned with their CT_max_ results (i.e. blackchin shiner had greater tolerance in both; Reemeyer  *et al.*, 2024). As stated above, little is known about the factors that govern agitation temperature and why patterns in agitation might differ from CT_max_. However, agitation is a behavioural metric that demonstrates how an individual interacts with the environment as temperatures increase (e.g. to seek thermal refugia), whereas CT_max_ is a physiological response related to mortality. Our study may have observed conserved thermal sensitivity (i.e. agitation) between species compared to the *Minielluss* species since *Ammocrypta* species have high niche conservatism, occupy similar microhabitat, and may have a similar behavioural response to seek thermal refugia. Daniels  (1989) hypothesized that the fossorial behaviour of sand darters could be related to the use of substrate as thermal refugia since they observed that water temperature was 10°C cooler 2 cm into the substrate.

### Mass effects

The study demonstrated no independent effect of mass on CT_max_ in the intra-specific eastern sand darter comparison. However, mass influenced eastern sand darter CT_max_ previously when comparing two northern populations (Firth  *et al.*, 2021). Additionally, mass influenced CT_max_ in the inter-specific comparison with larger individuals having a higher CT_max_. While a relationship was observed in Firth  *et al.*  (2021) and in the inter-specific comparisons, the range of sizes used in this intra-specific study was smaller and may have been too small to have elicited a mass effect. Similar to our findings, shortnose sturgeon (*Acipenser brevirostrum*) demonstrated a positive relationship with mass and thermal tolerance (Zhang  and  Kieffer, 2014). If *Ammocrypta* species follow the climate-induced trend in declining fish size (Audzijonyte  *et al.*, 2020; Queiros  *et al.*, 2024), they may experience a decrease in CT_max_ that puts them at greater risk with future water temperature increases. Future research is needed to confirm our findings given that the data were collected *in situ* across the growing seasons, and mass and acclimatization temperature were weakly correlated with increasing mass as the growing season progressed. Thus, the observed relationship between CT_max_ and mass could have been inflated, leading to the observed relationship.

## Conclusions

In this study, we present data demonstrating no intra-specific differences in CT_max_ or T_ag_; however, population was important in predicting T_ag_, as it appeared in all the top models. Additionally, inter-specific difference in CT_max_ were minimal with only one robust comparison (i.e. eastern and naked sand darter) across the five top models and no differences in T_ag_. Thus, assessing multiple thermal traits is important for understanding the potential vulnerability to environmental change for a genus and its species, as well as individual populations. By studying T_ag_ in addition to CT_max_, we were able to report how that population was important for predicting sub-lethal stress and that the studied species had similar responses to thermal stress at the sub-lethal level but not the lethal level. Thus, our study demonstrates that even species with high niche conservatism within a small clade can have differences in CT_max_ and CT_max_ plasticity. Nevertheless, further research is needed to understand the full degree of species differences and the drivers behind them.

## Supplementary Material

Web_Material_coag055

## Data Availability

Data associated with this study are publicly available on Figshare (https://doi.org/10.6084/m9.figshare.32254776).
