# Supplementary material for: Intra- and inter-specific differences of upper thermal limits in the sand darters (Percidae: Ammocrypta)
Source: Conserv Physiol. 2026 Aug 10;14(1):coag055. doi: 10.1093/conphys/coag055 (PMC13455357; doi:10.1093/conphys/coag055)
Supplement: Supplementary file 1 [file web_material_coag055.zip › Supplementary_materials_coag055.docx]

**Supplementary Table 1:** Site locations for collection of each Ammocrypta species for each sampling timepoint.

| **Species** | | **Sample Time** | | **Latitude** | | **Longitude** | | **River** | | **Closest City** |
| --- | --- | --- | --- | --- | --- | --- | --- | --- | --- | --- |
| N. Eastern | | June 3-6 | | 43.109759 | | -80.245445 | | Grand River | | Brantford, Ontario |
|  | | July 15-18 | | 43.109759 | | -80.245445 | | Grand River | | Brantford, Ontario |
|  | | August 12-14 | | 43.109759 | | -80.245445 | | Grand River | | Brantford, Ontario |
| S. Eastern | | April 12-13 | | 37.819823 | | -83.575132 | | Red River | | Pine Ridge, Kentucky |
|  | | May 30-31 | | 37.819823 | | -83.575132 | | Red River | | Pine Ridge, Kentucky |
|  | | August 15-16 | | 37.819823 | | -83.575132 | | Red River | | Pine Ridge, Kentucky |
| Naked | | April 18 | | 32.48043 | | -87.12691 | | Oakmulgee Creek | | Selma, Alabama |
|  | | April 19 | | 32.946372 | | -87.14038 | | Cahaba River | | Centreville, Alabama |
|  | | June 5 | | 32.946372 | | -87.14038 | | Cahaba River | | Centreville, Alabama |
| Southern | | April 20 | | 32.855966 | | -87.19811 | | Cahaba River | | Centreville, Alabama |
|  | | April 21 | | 32.774635 | | -87.273553 | | Cahaba River | | Centreville, Alabama |
|  | | June 6 | | 32.855966 | | -87.19811 | | Cahaba River | | Centreville, Alabama |
|  |  | |  | |  | |  | |  | |

**Supplementary Table 2:** Sample size corrected Akaike information criterion (AIC_c_) rankings of the intra-specific CT_max_ model selection. Models shown in bold text have ΔAIC_c_ <2 and are considered equally plausible. K defines the number of model estimate parameters, *wi* denotes the Akaike weigh giving the probability of the model being the correct model, and R^2^ denotes the conditional (marginal) R^2^ for each model with a random effect and adjusted R^2^ for models without a random effect.

| **Model** | **K** | **AIC_c_** | **ΔAIC_c_** | ***w_i_*** | **R^2^** |
| --- | --- | --- | --- | --- | --- |
| **Temp + (1\|SampleEvent)** | **4** | **317.22** | **0.00** | **0.27** | **0.91 (0.89)** |
| **Temp*Mass + (1\|SampleEvent)** | **6** | **317.53** | **0.31** | **0.23** | **0.91 (0.90)** |
| **Temp + Mass + (1\|SampleEvent)** | **5** | **317.71** | **0.49** | **0.21** | **0.91 (0.89)** |
| **Temp + Pop + (1\|SampleEvent)** | **5** | **319.21** | **1.99** | **0.10** | **0.91 (0.89)** |
| Temp*Mass | 5 | 319.57 | 2.35 | 0.08 | 0.90 |
| Temp*Mass + Pop + (1\|SampleEvent) | 7 | 319.72 | 2.49 | 0.08 | 0.91 (0.90) |
| Temp + Population + Mass + (1\|SampleEvent) | 6 | 319.86 | 2.64 | 0.07 | 0.91 (0.89) |
| Temp*Pop + (1\|SampleEvent) | 6 | 320.90 | 3.67 | 0.04 | 0.91 (0.90) |
| Temp | 3 | 321.28 | 4.05 | 0.04 | 0.90 |
| Temp*Pop + Mass + (1\|SampleEvent) | 7 | 321.30 | 4.08 | 0.04 | 0.91 (0.90) |
| Temp*Mass + Pop | 6 | 321.70 | 4.48 | 0.03 | 0.90 |
| Pop*Mass + Temp + (1\|SampleEvent) | 7 | 322.04 | 4.82 | 0.02 | 0.91 (0.89) |
| Temp + Mass | 4 | 322.98 | 5.76 | 0.02 | 0.90 |
| Temp + Pop | 4 | 323.39 | 6.17 | 0.01 | 0.90 |
| Temp*Pop | 5 | 323.79 | 6.57 | 0.01 | 0.90 |
| Temp*Population + Mass | 6 | 324.64 | 7.42 | 0.01 | 0.90 |
| Temp + Pop + Mass | 5 | 325.01 | 7.79 | 0.01 | 0.90 |
| Temp + Pop*Mass | 6 | 327.06 | 9.84 | 0.00 | 0.90 |

**Supplementary Table 3:** Sample size corrected Akaike information criterion (AIC_c_) rankings of the intra-specific T_ag_ model selection. Models shown in bold text have ΔAIC_c_ <2 and are considered equally plausible. K defines the number of model estimate parameters, *wi* denotes the Akaike weigh giving the probability of the model being the correct model, and R^2^ denotes the conditional and marginal (in brackets) R^2^ for each model with a random effect and adjusted R^2^ for models without a random effect.

| **Model** | **K** | **AIC_c_** | **ΔAIC_c_** | ***w_i_*** | **R^2^** |
| --- | --- | --- | --- | --- | --- |
| **Temp*Pop + (1\|SampleEvent)** | **6** | **445.18** | **0.00** | **0.37** | **0.81 (0.79)** |
| **Temp*Pop + Mass + (1\|SampleEvent)** | **7** | **446.01** | **0.83** | **0.24** | **0.81 (0.79)** |
| Temp + Mass + (1\|SampleEvent) | 5 | 447.78 | 2.60 | 0.10 | 0.77 (0.67) |
| Temp + (1\|SampleEvent) | 4 | 448.24 | 3.05 | 0.08 | 0.77 (0.66) |
| Temp*Mass + (1\|SampleEvent) | 6 | 448.97 | 3.79 | 0.06 | 0.77 (0.65) |
| Temp + Population + Mass + (1\|SampleEvent) | 6 | 449.57 | 4.39 | 0.04 | 0.78 (0.69) |
| Temp + Pop + (1\|SampleEvent) | 5 | 449.73 | 4.55 | 0.04 | 0.78 (0.69) |
| Temp*Pop | 5 | 450.42 | 5.23 | 0.03 | 0.79 |
| Temp*Mass + Pop + (1\|SampleEvent) | 7 | 450.87 | 5.69 | 0.02 | 0.78 (0.67) |
| Temp + Pop*Mass + (1\|SampleEvent) | 7 | 451.36 | 6.18 | 0.02 | 0.78 (0.70) |
| Temp*Population + Mass | 6 | 452.35 | 7.17 | 0.01 | 0.79 |
| Temp + Pop*Mass | 6 | 472.42 | 27.24 | 0.00 | 0.75 |
| Temp*Mass + Pop | 6 | 474.13 | 28.95 | 0.00 | 0.75 |
| Temp*Mass | 5 | 474.62 | 29.44 | 0.00 | 0.75 |
| Temp + Pop | 4 | 474.68 | 29.50 | 0.00 | 0.74 |
| Temp + Pop + Mass | 5 | 474.76 | 29.58 | 0.00 | 0.75 |
| Temp + Mass | 4 | 475.44 | 30.26 | 0.00 | 0.74 |
| Temp | 3 | 477.56 | 32.38 | 0.00 | 0.74 |

**Supplementary Table 4:** Sample size corrected Akaike information criterion (AIC_c_) rankings of the inter-specific species CT_max_ model selection. Models shown in bold text have ΔAIC_c_ <2 and are considered equally plausible. K defines the number of model estimate parameters, *wi* denotes the Akaike weigh giving the probability of the model being the correct model, and R^2^ denotes the conditional and marginal (in brackets) R^2^ for each model with a random effect and adjusted R^2^ for models without a random effect.

| **Model** | **K** | **AIC_c_** | **ΔAIC_c_** | ***w_i_*** | **R^2^** |
| --- | --- | --- | --- | --- | --- |
| **Temp*Species + Mass + (1\|Species:SampleEvent)** | **12** | **529.41** | **0.00** | **0.27** | **0.89 (0.89)** |
| **Temp*Species** | **10** | **530.47** | **1.06** | **0.16** | **0.88** |
| **Temp*Species + Mass** | **11** | **530.50** | **1.09** | **0.16** | **0.88** |
| **Temp*Species + (1\|Species:SampleEvent)** | **11** | **530.75** | **1.34** | **0.14** | **0.89 (0.89)** |
| **Temp + Species + Mass + (1\|Species:SampleEvent)** | **9** | **530.84** | **1.43** | **0.13** | **0.89 (0.87)** |
| Temp*Mass + Species + (1\|Species:SampleEvent) | 10 | 531.92 | 2.52 | 0.08 | 0.89 (0.87) |
| Temp + Species + (1\|Species:SampleEvent) | 8 | 532.24 | 2.83 | 0.07 | 0.89 (0.87) |
| Temp + Species*Mass + (1\|Species:SampleEvent) | 13 | 537.24 | 7.83 | 0.01 | 0.89 (0.87) |
| Temp + Species | 7 | 540.48 | 11.07 | 0.00 | 0.88 |
| Temp*Mass + Species | 9 | 541.26 | 11.85 | 0.00 | 0.88 |
| Temp + Mass + (1\|Species:SampleEvent) | 5 | 541.28 | 11.87 | 0.00 | 0.87 (0.75) |
| Temp + Species + Mass | 8 | 541.43 | 12.02 | 0.00 | 0.88 |
| Temp*Mass + (1\|Species:SampleEvent) | 6 | 542.15 | 12.74 | 0.00 | 0.88 (0.76) |
| Temp + Species*Mass | 12 | 545.00 | 15.59 | 0.00 | 0.88 |
| Temp + (1\|Species:SampleEvent) | 4 | 545.63 | 16.23 | 0.00 | 0.87 (0.72) |
| Temp*Mass | 5 | 635.18 | 105.77 | 0.00 | 0.81 |
| Temp + Mass | 4 | 639.62 | 110.22 | 0.00 | 0.81 |
| Temp | 3 | 654.53 | 125.12 | 0.00 | 0.79 |

**Supplementary Table 5:** Sample size corrected Akaike information criterion (AIC_c_) rankings of the inter-specific species T_ag_ model selection. Models shown in bold text have ΔAIC_c_ <2 and are considered equally plausible. K defines the number of model estimate parameters, *wi* denotes the Akaike weigh giving the probability of the model being the correct model, and R^2^ denotes the conditional and marginal (in brackets) R^2^ for each model with a random effect and adjusted R^2^ for models without a random effect.

| **Model** | **K** | **AIC_c_** | **ΔAIC_c_** | ***w_i_*** | **R^2^** |
| --- | --- | --- | --- | --- | --- |
| **Temp +( 1\|Species:SampleEvent)** | **4** | **716.96** | **0.00** | **0.34** | **0.69 (0.59)** |
| **Temp + Mass +( 1\|Species:SampleEvent)** | **5** | **717.25** | **0.29** | **0.30** | **0.70 (0.59)** |
| **Temp*Mass + (1\|Species:SampleEvent)** | **6** | **718.21** | **1.26** | **0.18** | **0.69 (0.58)** |
| Temp + Species + Mass + (1\|Species:SampleEvent) | 9 | 719.97 | 3.01 | 0.08 | 0.70 (0.64) |
| Temp*Mass + Species + (1\|Species:SampleEvent) | 10 | 721.64 | 4.68 | 0.03 | 0.70 (0.63) |
| Temp + Species + (1\|Species:SampleEvent) | 8 | 721.86 | 4.90 | 0.03 | 0.70 (0.62) |
| Temp*Species + Mass + (1\|Species:SampleEvent | 11 | 723.29 | 6.33 | 0.01 | 0.72 (0.67) |
| Temp*Species + (1\|Species:SampleEvent) | 11 | 723.29 | 6.33 | 0.01 | 0.71 (0.67) |
| Temp + Species*Mass + (1\|Species:SampleEvent) | 13 | 727.78 | 10.83 | 0.00 | 0.70 (0.64) |
| Temp*Species + Mass | 11 | 740.35 | 23.40 | 0.00 | 0.69 |
| Temp*Mass + Species | 9 | 741.08 | 24.12 | 0.00 | 0.68 |
| Temp + Species + Mass | 8 | 741.67 | 24.71 | 0.00 | 0.68 |
| Temp*Species | 10 | 741.92 | 24.97 | 0.00 | 0.68 |
| Temp + Mass | 4 | 745.30 | 28.34 | 0.00 | 0.67 |
| Temp | 3 | 745.66 | 28.70 | 0.00 | 0.66 |
| Temp*Mass | 5 | 746.08 | 29.12 | 0.00 | 0.67 |
| Temp + Species | 7 | 748.38 | 31.42 | 0.00 | 0.67 |
| Temp + Species*Mass | 12 | 748.55 | 31.59 | 0.00 | 0.68 |

**Supplementary Figure 1:** Temperature traces over the entire CT_max_ trial in minutes for eastern sand darter (red), Florida sand darter (black), southern sand darter (green), naked sand darter (purple), and scaly sand darter (yellow).

**Supplementary Figure 2:** Intra-specific differences in agitation temperature mean (A) and T_ag_ estimated acclimation response ratio (C) for T_ag_ model 2 and critical thermal maximum mean (B) for CT_max_ model 4 between northern (black) and southern (red) eastern sand darter (*Ammocrypta pellucida*) populations for linear mixed effect model at 23.5°C. Error bars represent the 95% confidence intervals.

**Supplementary Figure 3:** Inter-specific differences in critical thermal maximum mean (A, C, E, G) and estimated acclimation response ratio (B, D, F) for eastern (red), southern (green), scaly (yellow), naked (purple), and Florida (orange) sand darters (*Ammocrypta*) for linear mixed effect model two to five at 21.91°C for each species. Error bars represent the 95% confidence intervals.
